# Physical Activity Targeted at Maximal Lipid Oxidation: A Meta-Analysis

**DOI:** 10.1155/2012/285395

**Published:** 2012-08-14

**Authors:** A. J. Romain, M. Carayol, M. Desplan, C. Fedou, G. Ninot, J. Mercier, A. Avignon, J. F. Brun

**Affiliations:** ^1^EA 4556 Epsylon: Laboratory of Dynamics of human Abilities & Health Behaviors, University Montpellier 1, 34000 Montpellier, France; ^2^Department of Nutrition and Diabetes, University Hospital of Montpellier, 34295 Montpellier, France; ^3^INSERM U1046 “Physiologie et Médecine Expérimentale du Cœur et du Muscle”, Université Montpellier 1, Université Montpellier 2 et Centre Hospitalier Universitaire Lapeyronie, 34295 Montpellier, France; ^4^Department of Clinical Physiology (CERAMM), University Hospital of Montpellier, 34295 Montpellier, France

## Abstract

Exercise is recognized as a part of the management of obesity and diabetes. Various protocols of exercise are proposed for the management of obesity, diabetes, and other metabolic diseases. One of the strategies proposed by several authors is low intensity endurance training targeted at the level of maximal oxidation. Large series using this technique are lacking. Addressing this issue, we performed a meta-analysis of the studies on anthropometric measurements. From a database of 433 articles, 15 were selected, including 279 subjects with 6 different populations. Studies duration ranged from 2 months to 12 months. Concerning weight loss, in the intervention versus control analysis, five studies with 185 participants were included with a significant effect size favors exercise (*P* = 0.02) without significant heterogeneity (*I* ^2^ = 0.0%, *P* = 0.83). Further randomized controlled trials for comparing it with other exercise protocols and defining its dose effectiveness on large samples are needed.

## 1. Introduction

Exercise training is now widely recognized as a key component of the management of obesity [1, 2] and diabetes [3]. At the beginning of the 21st century, the description of a curve of lipid oxidation [4, 5] led to the hypothesis that endurance exercise in obesity and diabetes should be targeted at this level of maximal lipid oxidation in order to obtain an optimal effect on lipids [6]. This curve is derived from the crossover concept [7] which is a physiological theory of exercise explaining that at rest, as at low intensities of exercise, the substrate of energy that is preferentially oxidized is lipids whereas at the highest intensities, carbohydrates are preferentially oxidized. However, only a few studies have addressed this working hypothesis on exercise and obesity or diabetes, and the bulk of current literature does not take into account this concept [8, 9].

Despite the fact that most of these studies involve a little number of subjects and are performed over a short duration of time, it was interesting to review them in a meta-analysis. The aim of this meta-analysis was to give an overall picture of the effects of exercise training targeted on maximal lipid oxidation.

## 2. Material and Methods

### 2.1. Literature Search

We conducted a research on (1) Pubmed (2) ISI Web of Science from 1994 to 2012, and (3) we also manually searched articles on sciences direct database published from 1994 to 2012 in English or French languages. The year 1994 was chosen being the date of the publication on the crossover concept [7]. Further information about the crossover concept is provided in a recent review [10, 11]. We (4) also included proceedings of congress when data were available by asking values to corresponding authors. Words written to perform research were “lipoxmax,” “lipoxmax AND training,” “lipoxmax AND physical activity,” “lipoxmax AND exercise,” “fatmax,” “fatmax AND training”, “fatmax AND physical activity”, “fatmax AND exercise”, “maximal fat oxidation,” “maximal fat oxidation AND training,” “maximal fat oxidation AND exercise," and “maximal fat oxidation AND physical activity”. 

Articles were selected by three different investigators. Differences of opinion for inclusion were resolved by discussion.

### 2.2. Study Selection

Studies were included in the meta-analysis if they met the following criteria: (1) designed as randomized controlled trial or clinical trial, (2) maximal fat-oxidation point was the training method according to the described protocol, (3) participants were males or females affected by chronic diseases without age restriction, and (4) anthropometric measurements and cholesterol were defined as outcomes. If data were duplicated in more than one publication, only the most recent publication was included in the analysis. 

Studies were excluded if (1) it was on animal models, (2) the study design was cross-sectional, (3) there was no intervention, and (4) the intervention was on healthy participants.

### 2.3. Extraction and Classification of the Data

Descriptive data regarding author, year of publication, pathology, study sample characteristics, type of design, and duration of training protocol were extracted from all selected articles. Weight, waist measurement, fat mass, and serum cholesterol were the selected outcomes. 

### 2.4. Statistical Methods and Analysis

Because of heterogeneity among included studies, the effect estimates were pooled using a random effects model with the method of DerSimonian and Laird. Firstly, pre- and postintervention mean differences (and their associated standard errors) were pooled for each outcome. Secondly, another analysis consisted of results extracted from randomized controlled trials only to obtain a pooled standardized mean difference of intervention group versus control group. The second analysis was only achieved for the weight outcome as there were less than three included studies that reported mean differences for the other outcomes.

Statistical analyses were performed by using Stata software version 10 (StataCorp. 2007. *Stata Statistical Software: Release 10*. College Station, TX: StataCorp LP).

Heterogeneity was tested by using Cochran's chi-square test (Q) to assess the consistency of associations as usual in meta-analysis [12]. To quantify the extent of heterogeneity of this collection of studies, we estimated the between-study variance (*I²*). This *I²* statistics describes the proportion of total variance in effect estimates due to the heterogeneity among studies; homogeneous studies should have an *I²* value of 0. 

When there were sources of heterogeneity, meta regression was computed to test whether such factors as population, intervention duration, and training type (diet and exercise + diet) had an impact on the final pooled estimates and on the heterogeneity. When moderators were significant, we split the analyses according to this moderator. 

Publication bias was examined with the use of funnel plot which is a scatter plot of treatment effect against a measure of study size. The presented funnel plot includes pre- and postintervention mean differences of weight as it is the outcome for which we found the highest number of included studies.

## 3. Results

**Figure 1:**
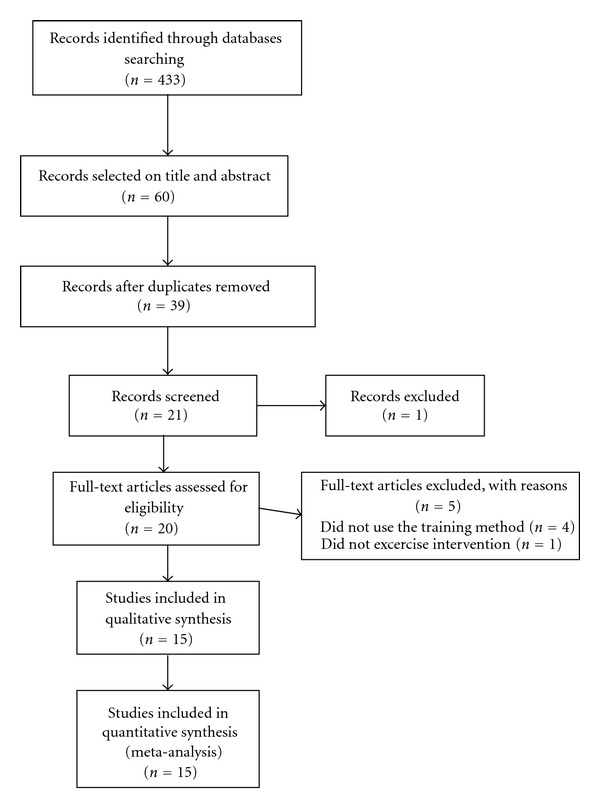
Flow chart of the study selection.

Researches using our different strategies within the different databases retrieved 433 articles. A sum of 60 articles were selected on the basis of their title and abstract, then a total of 15 articles [13–28] met the inclusion criteria and were thus included in the meta-analysis. Flow chart of the study selection is available in Figure 1. Descriptive characteristics of the studies are presented in Table 1. There were 3 randomized controlled trials, 2 controlled trials, and 10 clinical trials or random trials. A total of 279 subjects were recruited through the 15 studies. We found 6 different populations. Those populations were obese adolescents [13–15, 18, 21, 26, 27] and nondiabetic obese adults [24], patients with metabolic syndrome [16, 19], patients affected by human immunodeficiency virus (HIV) [17], patients with type 2 diabetes (T2D) [20, 22, 25, 28], and patients with neuroleptic treatment [23]. The study with the highest sample is a randomized controlled trial including 63 T2D [28].

Several types of protocols were identified. There were studies with only training [13, 14, 16–28], studies with diet + training [13, 15, 18, 21, 26].

Duration of studies varied from 2 months to 12 months. The study that included people affected by HIV presented the longest followup [17].

Number of training sessions per week ranged from 2 sessions of 45 minutes to 4 sessions of 90 minutes per week. Two studies [17, 23] did not precise the number of sessions per week. One study used an incremental protocol [24] and one an exercise protocol where the numbers of sessions were progressively reduced [14]. The whole studies used ergometer as training material during their sessions.

Concerning the nutritional intervention, the whole studies used hypocaloric diet. One studies set −500 kilocalories (Kcal) per day below the energy requirements at time of the study without precision about the repartition of nutriments [21]. Three others studies set −500 Kcal per day with 15% from proteins, 55% from carbohydrates, and 30% from lipids [13, 18, 26], and 1 study set −300 Kcal per day with meal composed of 15% from proteins, 55% from carbohydrates and 30% from lipids [15].

### 3.1. Before versus after Intervention

#### 3.1.1. Weight

In included studies, loss of weight varied from 0 Kg to 11.5 Kg and 11 studies reported a significant loss between before and after intervention. 

**Figure 2:**
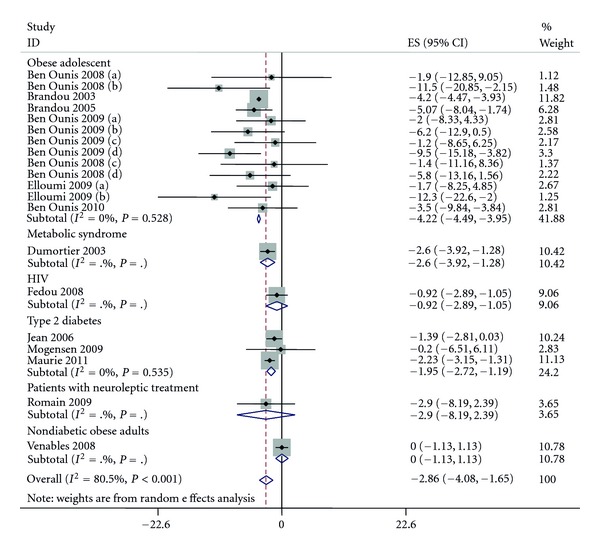
Pooled standard errors and confidence intervals of weight loss in before to after design.

The pooled effect estimate and its associated 95% confidence interval (CI) of weight loss of after versus before intervention was −2.86 Kg (95% CI: −4.07; −1.64) (see Figure 2). The analysis showed a significant heterogeneity among studies (*P* < 0.0001, *I*² = 82%).

#### 3.1.2. Fat Mass

In included studies, loss of fat mass varied from −0.01 Kg to −12.1 Kg and 10 studies reported a significant loss between before and after intervention.

**Figure 3:**
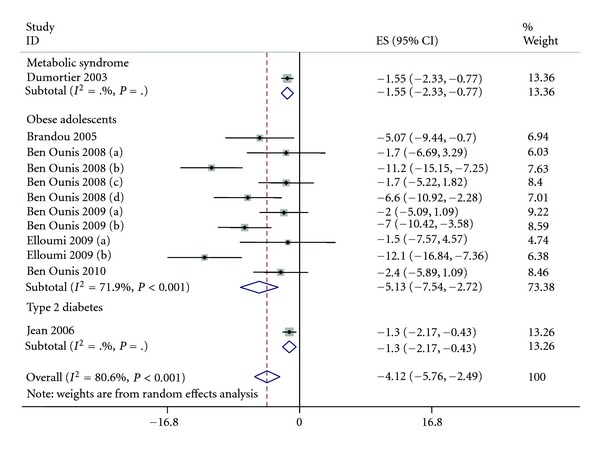
Pooled standard errors and confidence intervals of fat mass loss in before to after design.

The pooled effect estimate of fat mass loss of after versus before intervention was −4.1 Kg (95% CI: −5.8; −2.3) (*P* < 0.0001) (see Figure 3). The analysis showed a significant heterogeneity among studies (*P* < 0.0001, *I*² = 81%).

#### 3.1.3. Waist Circumference

In included studies, changes in waist circumference varied from −2.9 cm to −12.3 cm and 7 studies reported a significant decrease between before and after intervention.

**Figure 4:**
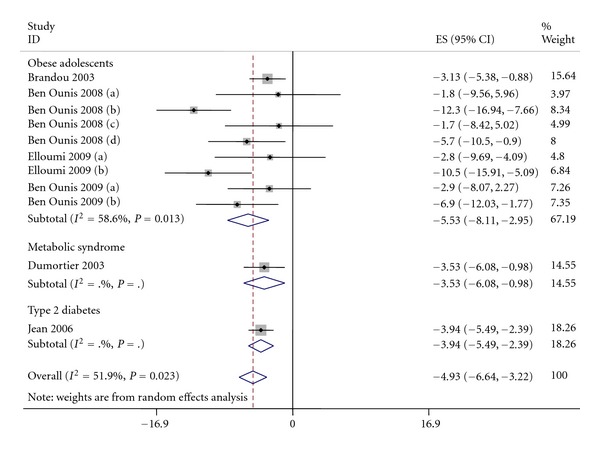
Pooled standard errors and confidence intervals of waist circumference in before to after design.

The pooled effect estimate of waist circumference change of after versus before intervention was −4.9 cm (95% CI: −6.6; −3.2) (*P* < 0.0001) (see Figure 4). The analysis showed a significant heterogeneity among studies (*P* = 0.02, *I*² = 52%).

#### 3.1.4. Cholesterol

In included studies, changes in total cholesterol varied from 0 mmol/L to −0.66 mmol/L and 3 studies reported a significant change between after and after intervention. The pooled effect estimate of total cholesterol of after versus after intervention was −0.26 mmol/L (95% CI: −0.35; −0.17) (*P* < 0.0001). The analysis did not show any significant heterogeneity (*P* = 0.18).

### 3.2. Intervention versus Control

Due to lack of data for fat mass, waist circumference and cholesterol, intervention versus control analysis was only computed for weight.

5 studies which involved 185 participants (128 in the intervention group and 57 in the control group) were included in the analysis. All 5 studies reported significant loss of weight in intervention group compared to control group.

**Figure 5:**
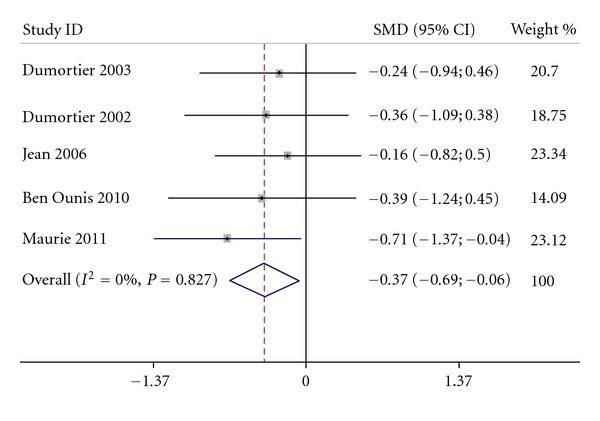
Pooled standardized mean difference and confidence intervals of weight comparing intervention versus control.

The pooled standardized mean difference of intervention versus control was −0.37 (95% CI: −0.69; −0.06) (*P* = 0.02) and favors intervention (see Figure 5). The analysis did not show significant heterogeneity (*P* = 0.83, *I*² = 0.0%).

### 3.3. Analyses of Moderators

All results concerning the moderators are in Table 2.

#### 3.3.1. Weight

Concerning weight loss, the metaregression showed that population (*P* < 0.001) and nutrition intervention (*P* < 0.01) were significant moderators of weight loss while duration of intervention was not (*P* = 0.227).

As population was a significant moderator, we thus split the results according to population (see Figure 2). These secondary analyses showed that results remained significant in obese adolescent (−4.21 (95% CI: −4.48; −3.94)) with no heterogeneity (*P* = 0.52, *I*² = 0.0%), metabolic syndrome (−2.60 (95% CI: −3.92; −1.27)), and heterogeneity was not applicable due to the number of included studies (*n* = 1), T2D (−1.95 (95% CI: −2.72; −1.18)) with no heterogeneity (*P* = 0.53; *I*² = 0.0%).

Splitting the results according to an intervention in nutrition or not, the mean difference remained significant for intervention only with exercise (−1.95 (95% CI: −3.28; −0.62)) and was larger with interventions including diet + exercise (−6.81 (95% CI: −9.15; −4.47)), without overlap between the CI showing a significant difference between the two interventions. The heterogeneity remained present for intervention without nutrition (*P* < 0.001, *I*² = 85%) but not in those included both diet and exercise (*P* = 0.54, *I*² = 0.0%).

#### 3.3.2. Fat Mass

Concerning this outcome, the metaregression showed that neither population (*P* = 0.19) nor intervention duration (*P* = 0.55) were significant moderators. Only nutrition remained significant (<0.001).

Thus, separating population according to presence or not of nutritional intervention, the mean difference was significant for interventions that only considered exercise (−1.49 (95% CI: −2.04; −0.94)), and was also larger for intervention including diet and exercise (−8.32 (95% CI: −10.89; −5.74)). As for weight, no overlap was founded between the CI signifying a significant difference between the interventions. There was no heterogeneity in exercise interventions (*P* = 0.99, *I*² = 0.0%) as for diet + exercise (*P* = 0.10, *I*² = 48.6%).

#### 3.3.3. Waist Circumference

The metaregression showed that population was not a significant moderator (*P* = 0.51), neither was intervention duration (*P* = 0.36). Only nutrition remained a significant moderator (*P* = 0.004) of waist circumference.

Thus, considering the presence of nutrition or not, the effect of exercise was still significant (−3.51 (95% CI: −4.58; −2.43)) as diet + exercise (−8.87 (95% CI: −11.98; −5.75)) without significant difference due to the overlap between the CI. Then, no heterogeneity was noted either for exercise only (*P* = 0.98, *I*² = 0.0%) as for diet + exercise (*P* = 0.19, *I*² = 36%).

#### 3.3.4. Cholesterol

As there was no heterogeneity in the meta-analysis (*P* = 0.02,   *I*² = 52%), we did not analyze moderators of cholesterol.

#### 3.3.5. Publication Bias

**Figure 6:**
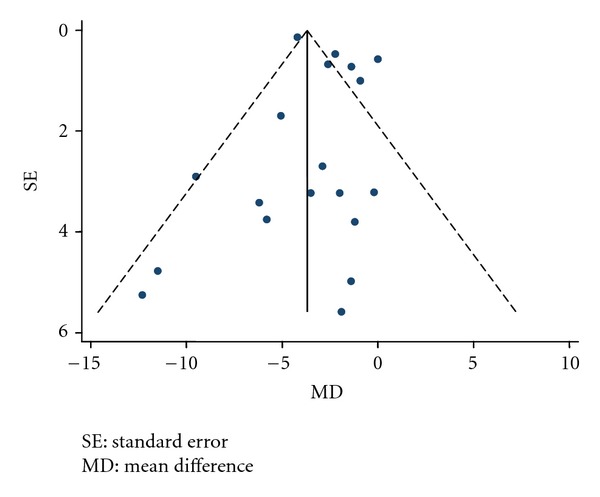
Funnel plot of weight and its 95% confidence interval in before to after design.

We looked for a publication bias for weight loss between before and after intervention using funnel plot representation (Figure 6). In absence of publication bias, the studies' results should be symmetrically placed about the line that represent the effect estimate to form a shape of “funnel,” taking into account that the results from smaller the smaller studies would be more widely spread around the average effect because of their larger standard errors. On Figure 6, studies are equally spread around the average effect; however, we can observe a weak tendency for the smaller studies to give more important weight loss that the larger studies which can suggest a possibility of publication bias. It would signify that there are other small studies which have been carried out but which have not been published and that those included in this meta-analysis are biased in favor of weight loss. However, we cannot make the conclusion of the presence of a publication bias because this distribution may also arise from the small number of included studies.

## 4. Discussion

This meta-analysis confirms the conclusions of the individual studies, that are very low intensity training targeted at the level of maximal fat oxidation significantly decreases body weight, fat mass, waist circumference and total cholesterol. On the average, the effects of this variety of training are thus well confirmed, and their average magnitude is more precisely described. 

Some methodological aspects of our meta-analysis need to be discussed. First, only 5 studies include a control (nonexercising) group. Actually, we found more studies testing the effect of this method versus caloric restriction group than versus a control nonexercising group. Although all the 5 studies including a control group evidenced a superiority of intervention versus control, the pooled estimate shows a medium size effect on weight loss (−0.37) for the intervention group compared to the control group. This effect size could arise from a lack of power due to the low sample size: 128 intervention subjects versus 57 control subjects. However, if these results may seem low, they can be compared with the meta-analysis from Wu et al. [29]. In their meta-analysis, they compared the effect of diet + exercise versus diet only on weight loss and they obtained a standardized mean difference of −0.25 (95% CI: −0.36; −0.14) in favor of diet + exercise whereas we obtained a standardized mean difference of −0.37 (95% CI: −0.69; −0.06) in favor of exercise intervention. These results showed that specific physical activity training at the level of maximal lipid oxidation could give results similar to those of other intervention even if these results should be interpreted carefully because the intervention was not identical. 

The number of studies and especially of randomized controlled trials also requires comments. This number is quite small. Moreover, sample sizes in each study are relatively reduced, as they varied from 6 to 39 subjects. Therefore, there is a lack of large studies and especially of large randomized controlled trials for this variety of training. Another issue we have to discuss is the heterogeneity found in several analyses. Significant heterogeneity was detected for weight, fat mass and waist circumference comparing pre- and postintervention results. This heterogeneity could arise from the heterogeneity of included populations; indeed, participants suffer from various diseases: obesity [24], metabolic syndrome [16, 19], HIV-1 infection [17], T2D [20, 22, 25, 28], and psychiatric diseases [23]. In addition, participants present heterogeneous ages: some are adolescents, others are young adults and others are older adults. This source of heterogeneity was confirmed by the meta-regression showing that the population was a significant moderator of the results and when we separated the analysis according to the population, the heterogeneity became non-significant. So, the absence of heterogeneity with significant results according to population reinforced the interest of training at maximal lipid oxidation throughout different populations with chronic diseases. However, population was only a significant moderator for weight. Another source of heterogeneity is the type of interventions that differed from a study to another. Indeed, several studies only proposed exercise to their intervention group whereas others included exercise as well as nutrition management. Furthermore, the analysis of metaregression showed that nutrition was a significant moderator for each studied outcome. We can observe that studies reported better results when diet is associated to exercise as evidenced by the 95% CI showing significant differences in weight and fat mass but not for waist circumference. The synergistic effect of diet and exercise in obesity is now well established [1], and meta-analyses show that exercise by its own improves the effect of diet [29]. However, the effect of diet and the effect of exercise are difficult to delineate and results from our meta-analysis concerning the nutrition should be interpreted in light of the fact that nutrition interventions were all hypocalorics.

Concerning the duration of interventions, it was not (as it could have been expected) a significant moderator. This could be explained by the fact that only the study from Fédou et al. [17] reported a duration superior to 3 months. 

Interestingly, some studies [26] demonstrated an important average weight loss (8 kg over two months) with a protocol based on 90 min/day exercise at the level of maximal lipid oxidation. This could suggest that large weekly volumes of exercise training may be much more efficient than those used usually (i.e, 3 × 45 min/week). Studies on the dose-efficiency of this training procedure remain to be performed. 

Actually, most of these studies used a moderate weekly amount of exercise (in most papers 135 min per week) according to the guidelines available in the early 2000s. It is interesting to point out that such a moderate training protocol has demonstrable metabolic effects, as evidenced by two biopsy studies [20, 30]. An improvement in mitochondrial oxidation can be observed after only 2 months of training targeted on maximal lipid oxidation at only 90 min/wk, and is correlated to an increase in the ability to oxidize lipids at exercise [20, 30–32]. This effect of training at the level of maximal lipid oxidation on the ability to oxidize lipids at exercise is demonstrated in all studies including this measurement. Therefore it is clear that the ability to oxidize lipids at exercise is increased by this kind of targeted training. 

Whether it also modifies resting energy expenditure and resting lipid oxidation and make training more effective over 24 h remains to be studied. 

The central questions about such protocols targeted at lipid oxidation levels are as the following: (1) does targeting training at this level of lipid oxidation improve results compared to more standard procedures or not; (2) is the energy deficit the only factor of the therapeutic effect of exercise or does targeting it on lipids make it more efficient? The debate is sometimes passionate but we think that both questions are not yet resolved and deserve careful consideration. 

Personal targeting of exercise training is a classic issue in respiratory diseases so that some guidelines recommend it [33] on the basis of studies showing its superiority [34]. However other guidelines consider that there is no clear advantage of targeting and that standard procedures are efficient enough [35]. In heart or lung diseases the logic level for targeting is the ventilatory threshold (*V*_*T*_) because it is related to dyspnea which is a major symptom in these pathologies. Personalized targeting at the *V*_*T*_ has also been proposed in diabetes [36] and proven a marked efficacy on the cost of diabetes treatment [37]. However, dyspnea being not a key symptom in obesity or diabetes, it was logic to propose a model of training based on a more metabolic parameter and the level of maximal lipid oxidation has been logically proposed for this purpose [1, 38, 39].

Concerning obesity and exercise, there is literature saying that “fat loss depends on energy deficit only, independently of the method for weight loss” [40]. There is also a dose-response relationship between the crude amount of exercise expressed in METs and the loss of abdominal fat [41]. However few recent studies suggest that the ability to oxidize lipids may explain the interindividual variability of the efficiency of exercise-based weight reduction procedures, so that individuals oxidizing more lipids at rest [42, 43] or during exercise [44, 45] respond better to exercise. Since exercise may have both orexigenic and satietogenic effects [46] (in trained and healthy participants), it can also be assumed that lipid oxidizing exercise is less orexigenic because it minimizes carbohydrate waste that occurs at higher intensities and may increase appetite. Accordingly, the weight-reducing effect of exercise targeted at the LIPOXmax may be mediated in part by alterations in food intake [47]. Although this issue remains conflictual, it is logic to investigate this question more thoroughly. At this time our meta-analysis of available data is unable to address this question. 

Studies comparing training targeted on lipid oxidation with other training procedures are scarce. Comparison with aerobic interval training [28] suggests that the latter exhibits stronger effects on aerobic capacity, blood pressure, and blood lipids, while training targeted on lipids induces a greater fat loss and a better improvement of blood glucose levels in diabetics. There is also an interesting study that has not been included in this meta-analysis because it is not individually targeted at the LIPOXmax, but which aims at comparing endurance training at 40% VO_2_max and 60% VO_2_max. The level of 40% VO_2_max was chosen because it was expected to elicit a maximal level of lipid oxidation [48]. This study evidences a two-fold higher fat mass loss at 40% compared to 60% VO_2_max, and thus suggests that endurance training in the zone of lipid oxidation is more efficient than endurance at a higher power intensity to decrease fat mass. Clearly this issue requires more investigation.

If an interesting efficiency of exercise training targeted on lipid oxidation, as suggested by this meta-analysis, were further demonstrated, this method would appear rather attractive because the exercise test used for the targeting is easy to perform and does not require a maximal stress which is not always safe in obese or diabetic patients. In most countries a test before exercise training is required to ensure the safety of the procedure and is most of the time also used for targeting. In obese and/or diabetic subjects exercise calorimetry during a submaximal-graded exercise test could be one of the procedures employed for this purpose.

In conclusion, this meta-analysis shows that training targeted at maximal fat oxidation (mostly used 3 times a week) decreases fat mass and body weight and improves blood cholesterol. This method seems thus to be interesting in chronic diseases such as obesity and diabetes, but this evidence is based on small size studies and a host of issues remain to be clarified. Mostly, large-scale randomized controlled trials are needed to confirm these findings.

## Figures and Tables

**Table 1:** Descriptive characteristics of the included studies.

First author	Year	Sample size	Population	Protocol	Number of sessions/week	Duration (months)	Weight (Kg)	Waist circumference (cm)	Fat mass (Kg)	Cholesterol (mmol/L)
Ben Ounis [13]	2008	8	ObeAdo	REE	4 sessions of 90 min/week	2	−1.9	−1.8	−1.7	−0.21
		8	ObeAdo	REE + diet	4 sessions of 90 min/week	2	−11.5	−12.3	−1.2	−0.51
Brandou [14]	2003	14	ObeAdo	REE	7 sessions/week during 2 weeks. Then 1/week during 6 weeks	2	−3.72	−3.73		0.04
Brandou [15]	2005	7	ObeAdo	REE + diet	2 sessions of 35 min/week	2	−5.2		−5.07	
Dumortier [16]	2003	28	MetSyn	REE	3 sessions of 40 min/week	2	−2.6	−3.53	−1.4	
Fédou [17]	2008	10	HIV	REE	Not precised	12	−0.92		−0.01	−0.28
Ben Ounis [18]	2009	18	ObeAdo	REE	4 sessions of 90 min/week	2	−2	−2.9	−2	
		18	ObeAdo	REE + diet	4 sessions of 90 min/week	2	−6	−6.9	−7	
Dumortier [19]	2002	21	Obese	REE	3 sessions of 45 min/week	2				0
Bordenave[20]	2008	11	T2D	REE	2 sessions of 45 min per week	2.5			−3.13	
Ben Ounis [21]	2009	9	ObeAdo	REE	4 sessions of 90 min/week	2	−1.2		−1.4	
		9	ObeAdo	REE + diet	4 sessions of 90 min/week	2	−9.5		−5.9	
Jean [22]	2006	28	T2D	REE	3 sessions of 45 min/week	3	−1.3	−3.94	−0.66	−0.01
Romain [23]	2009	17	Psychiatry	REE	not precised	3	−2.9			
Venables [24]	2008	8	Obese	REE	5 sessions of 30 min/week with an incrementation up to 60 min/week	2 × 4 weeks	−0.2		−0.1	
Mogensen [25]	2009	12	T2D	REE	5 sessions of 30 min/week	2.5	0.2	−2.8		
Elloumi [26]	2009	7	ObeAdo	REE	4 sessions of 90 min/week	2	−1.7	−10.5	−1.5	
		7	ObeAdo	REE + diet	4 sessions of 90 min/week	2	−12.3		−12.1	
Ben Ounis [27]	2010	32	ObeAdo	REE	4 sessions of 90 min per week	2	−4.7	−8	−2.8	
Maurie [28]	2011	39	T2D	REE	3 sessions of 45 min/week	3	−2.23			

ObeAdo: obese adolescent, MetSyn: metabolic syndrome, T2D: type 2 diabetes, HIV: human immunodeficiency virus, and REE: training.

Weight, waist circumference, fat mass, and cholesterol are delta values between the beginning and the end-point of the studies.

**Table 2:** Results from analyses of moderators.

Moderators	Beta	Standard error	*P* value	95% CI
Weight				
Population	1.05	0.12	<0.001	0.81; 1.29
Duration	0.25	0.20	0.23	−0.17; 0.67
Nutrition	−5.09	1.47	0.003	−8.18; −2.01
Fat mass				
Population	2.19	1.57	0.19	−1.32; 5.70
Duration	1.79	2.93	0.55	−4.74; 8.34
Nutrition	−6.75	0.97	<0.001	−8.92; −4.58
Waist circumference				
Population	0.95	1.38	0.51	−2.80; 4.90
Duration	2.07	2.16	0.36	−2.80; 6.96
Nutrition	−5.37	1.38	<0.001	−8.49; −2.25

95% CI: 95% confidence intervals.
